# Tailoring plant-associated microbial inoculants in agriculture: a roadmap for successful application

**DOI:** 10.1093/jxb/eraa111

**Published:** 2020-03-11

**Authors:** Maged M Saad, Abdul Aziz Eida, Heribert Hirt

**Affiliations:** 1 DARWIN21, Biological and Environmental Sciences and Engineering Division, King Abdullah University of Science and Technology (KAUST), Thuwal, Saudi Arabia; 2 Institute of Plant Sciences Paris-Saclay (IPS2), Gif-sur-Yvette Cedex, France; 3 Max F. Perutz Laboratories, University of Vienna, Vienna, Austria; 4 University of Edinburgh, UK

**Keywords:** Abiotic and biotic stress, DARWIN21, desert bacteria, endophytes, plant growth-promoting rhizobacteria (PGPRs), plant microbiome, plant–microbe interaction, soil microbial community, synthetic community (SynCom)

## Abstract

Plants are now recognized as metaorganisms which are composed of a host plant associated with a multitude of microbes that provide the host plant with a variety of essential functions to adapt to the local environment. Recent research showed the remarkable importance and range of microbial partners for enhancing the growth and health of plants. However, plant–microbe holobionts are influenced by many different factors, generating complex interactive systems. In this review, we summarize insights from this emerging field, highlighting the factors that contribute to the recruitment, selection, enrichment, and dynamic interactions of plant-associated microbiota. We then propose a roadmap for synthetic community application with the aim of establishing sustainable agricultural systems that use microbial communities to enhance the productivity and health of plants independently of chemical fertilizers and pesticides. Considering global warming and climate change, we suggest that desert plants can serve as a suitable pool of potentially beneficial microbes to maintain plant growth under abiotic stress conditions. Finally, we propose a framework for advancing the application of microbial inoculants in agriculture.

## Teaser

Successful application of plant-associated microbial inoculants for sustainable agriculture requires a holistic approach of examining and understanding the soil environment, crop of interest, and associated microbial communities.

## Introduction

According to the United Nations Organization, the current world population of 7.6 billion is expected to increase beyond 9.8 billion by the year 2050 (United Nations, 2017). Accompanying this dramatic growth in population is the anticipated increase in the demand for agricultural food and feed products and the evident rise in environmentally destructive human activities, such as deforestation and the overuse of chemical fertilizers and pesticides in agriculture. The continuous deforestation, industrialization, and excessive use of fossil fuels have escalated the rise of CO_2_ concentrations in the atmosphere, leading to higher greenhouse gas emissions and average global temperatures (Mgbemene et al., 2016). Subsequently, these activities and phenomena have led to reductions in cultivatable land and crop productivity. Furthermore, the scarcity of freshwater resources or its inaccessibility and the high costs of water treatment and desalination further present a challenge to meet water demand for the agriculture sector (Beltrán and Koo-Oshima, 2006; Rosegrant et al., 2009). The combination of all these problems and challenges poses a serious threat to global food security and stability of economies, especially in developing countries.

The solution to those challenges necessitates multiple approaches, including the use of plant growth-promoting microbes as biostimulants to increase crop productivity. The concept of using biostimulants in agriculture is not new, and application of microbial consortia or single microbes as inoculum was previously addressed (Kong et al., 2018; Woo and Pepe, 2018). However, the successful transfer of microbial inoculants from the lab to the field remains a challenge. This is primarily due to the presence of many crop species and crop varieties, variable environmental conditions between fields, and the exponential increase in the number of microbial isolates. Therefore, a holistic approach towards the use of ‘biostimulants’ is needed via ‘diagnostics’ of the field environment (e.g. soil) and desired crop (e.g. genotype), selecting best agricultural practices, screening for inoculants from available culture collections, increasing scientific research in the field of microbiomes, and, finally, incorporating all the latter into large-scale industrial production and field application (Mitter et al., 2019; Pascale et al., 2019).

In this review, we will shed light on the different biotic (e.g. plants or pathogens) and abiotic (e.g. soil or climate) factors shaping microbial communities in the soil, rhizosphere, and plant. The limitations and complexities of microbial community experiments and their applications in agriculture will also be discussed. Finally, a roadmap will be presented for the successful application of microbial inoculants in agriculture.

## We are not alone: the concept of holobiont and plant-associated microbiota

Plants, animals, and almost all multicellular organisms are no longer considered as standalone individual organisms. Instead, they co-exist and are in constant interactions with their surrounding biota (Margulis and Fester, 1991; Turnbaugh et al., 2007; Bosch and McFall-Ngai, 2011). In the late 19th century, Karl Möbius named this interaction or co-existence as ‘biocenosis’ or ‘living community’ (Möbius, 1877). In 1991, Lynn Margulis proposed the term ‘Holobionts’—Holo is derived from the ancient Greek word ȍλος (hólos) for ‘whole’. Margulis described that any physical association between individuals of different species for a significant part of their life span is termed symbiosis and all participants in the symbiotic interaction are symbionts (Margulis and Fester, 1991; Bordenstein and Theis, 2015). A strictly microbe-dependent lifestyle has profound evolutionary consequences and suggests that the phenotype of a healthy host cannot be explained exclusively by its genome (Bosch and McFall-Ngai, 2011).

The advent of next-generation sequencing (NGS) opened up possibilities to study these close interactions between a host—human, animal, or plant—and its associated microbial community (Bosch and Miller, 2016; Greer et al., 2016; Sender et al., 2016). In addition, NGS can provide evidence for an active dialogue within the holobiont (host and associated microbiota) in coordinating and synchronizing signaling pathways and metabolic activities for maintaining a long-term, healthy co-existence (Rosenberg et al., 2010; Wier et al., 2010; Walter et al., 2011; Gilbert et al., 2012). Biological signals within the holobiont ecosystem could function as ‘Zeitgebers’ or time tuners (Leone et al., 2015; Lee et al., 2019). For example, signaling molecules produced by gut microbes were required for the functioning of the circadian clock in the host intestinal epithelial cells (Mukherji et al., 2013; Leone et al., 2015). Other living organisms, such as insects and plants, carry symbiotic microbes that provide defense against natural enemies (Arnold et al., 2003; Jaenike et al., 2010).

**Fig. 1.**
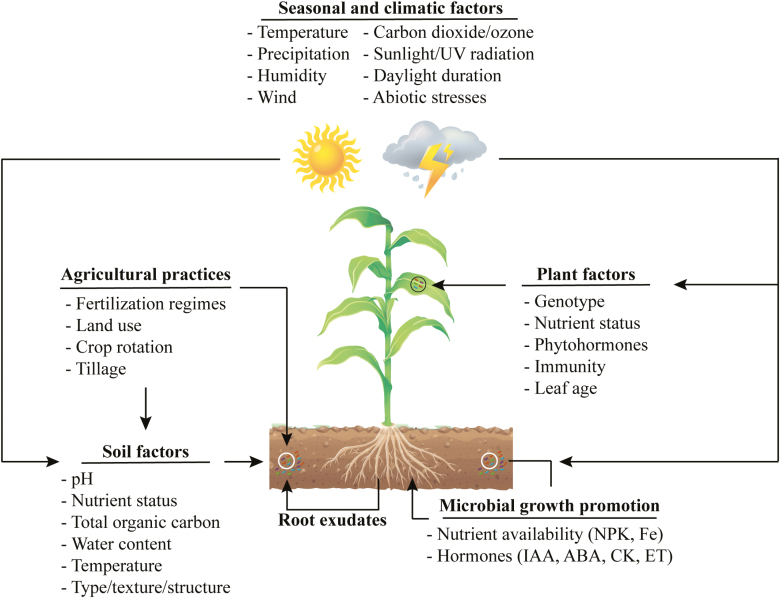
Microbial communities are shaped by several factors that must be considered in agricultural applications. Seasonal and climatic factors alter soil physicochemical properties and plant physiology. Microbial communities in the soil are affected by seasonal and climatic factors and soil factors. Plant factors alter microbial communities in the phyllosphere, endosphere, and rhizosphere, with the latter being via root exudates. Agricultural management practices can cause changes in the microbial communities in the soil either directly or via altering soil properties. Microbes associated with plants, in either the rhizosphere or the endosphere, are capable of promoting plant growth by making nutrients available or producing/modulating phytohormones.

Advances in NGS and culture-independent methods demonstrated that terrestrial plants are heavily colonized by a wide diversity of microorganisms, including bacteria, fungi, oomycetes, and protozoa (Kemen, 2014; Bulgarelli et al., 2015; Hacquard et al., 2015). Plants accommodate and interact with different microbes (Fig. 1) within their tissues (endosphere); they also interact with the surrounding microbial community present in the narrow region of soil surrounding the root system (rhizosphere) and around the stems, leaves, flowers, and fruits (phyllosphere). It is also clear now that microbiota play a major role in plant health and fitness (Müller et al., 2016). These microbes can colonize different plant organs either inside (endophytic) or attached to the surface (ectophytic).

The last two decades saw a steady increase in the number of studies investigating microbial communities of both above- and below-ground plants species. In the model plant Arabidopsis (*Arabidopsis thaliana*), a core microbial community was identified, where the bacterial community and function in the leaves overlapped with those in the roots (Lundberg et al., 2012; Bodenhausen et al., 2013; Bai et al., 2015). Similar studies were also shown for crop plants such as lettuce (*Lactuca sativa*) and tomato (*Solanum lycopersicum*) (Ottesen et al., 2013), wild and domesticated barley (*Hordeum vulgare*) (Bulgarelli et al., 2015), and maize (*Zea mays*) under field conditions (Peiffer et al., 2013) and in the greenhouse (Rastogi et al., 2012; Williams and Marco, 2014). Several pioneer desert plants such as *Agave*, *Atriplex*, *Tribulus*, *Panicum*, *Euphorbia*, and *Zygophyllum* were also studied (Kaplan et al., 2013; Coleman-Derr et al., 2016; Eida et al., 2018). All the aforementioned studies collectively agree that the plant root endosphere is dominated by a small number of bacterial lineages, with Actinobacteria, Bacteroidetes, and Proteobacteria being the dominant phyla when compared with soil and rhizosphere bacterial communities. Nevertheless, the relative abundances of individual phyla or genera are dependent on multiple physical, chemical, and biological factors.

In almost all ecosystems, multidimensional interactions exist between microbes and their hosts, and these are governed by biotic and abiotic factors. Biotic factors are the living components of an ecosystem, such as microbes, insects, plants, and animals. Abiotic factors are the non-living chemical and physical parts of the environment and are commonly affected by time (day/night) and seasonal or climate changes, such as soil chemical and physical properties, temperature, UV levels, precipitation (rainfall), and CO_2_ levels (Fig. 1). Abiotic factors, including stresses such as drought, soil salinity, or extreme temperatures, are very complex and affect the physiochemical properties of both the soil and plants, and their associated microbial communities.

## The soil dictates which microbes are accommodated by a host plant

Soil represents a highly complex system comprising a variety of environments with different physical, chemical, and biological properties. It is one of the largest reservoirs of microbial biomass and diversity, and thus serves as a pool for recruitment of microbes and enrichment of root endophytic communities (Whitman et al., 1998; Hartman et al., 2008; Bulgarelli et al., 2012; Yeoh et al., 2017). The soil microbial community structures, functions, and compositions are susceptible to physical (e.g. soil structure), chemical (e.g. nutrient content), and biological (e.g. presence of pathogenic or beneficial microbes) changes in their surroundings (Fig. 1; Table 1) (Truog, 1947; de Vries et al., 2012; Fierer et al., 2012). High-throughput molecular techniques coupled with NGS have enhanced our ability to characterize prokaryotic (e.g. bacterial) and eukaryotic (e.g. fungal) communities in the soil in terms of taxonomic and phylogenetic structure, enzymatic activity, microbial function, and abundance and composition. Various factors influence soil microbial communities including pH, nutrient (e.g. carbon, nitrogen, phosphorus) content and availability, water/moisture content, temperature, and soil type, texture, and particle size.

**Table 1.** Soil factors that are responsible for shaping microbial communities

Factors	Summary	References
Soil and abiotic factors	pH can alter the solubility and availability of nutrients influencing microbial diversity and composition with stronger influence on bacteria than fungi.	Fierer and Jackson (2006); Lauber et al. (2008); Lauber et al. (2009); Shen et al. (2013); Maestre et al. (2015); Zhang et al. (2017); Shen et al. (2018)
	Soil fertilization (e.g. NPK) and soil amendment (e.g. carbon) practices can affect nutrient status and influence bacterial and fungal communities in soil; C content is important for microbial growth and survival	Marschner et al. (2004); Eilers et al. (2010); Goldfarb et al. (2011); Kuramae et al. (2012); Siciliano et al. (2014); Maestre et al. (2015); Francioli et al. (2016)
	Changes in temperature and water content (or precipitation) can affect soil pH and nutrient status, and influence microbial community composition and function	Pettersson and Bååth (2003); Habekost et al. (2008); Bárcenas-Moreno et al. (2009); Bell et al. (2009); Castro et al. (2010); Koranda et al. (2013); Zhang et al. (2019)
	Soil type, texture, structure, and particle size can affect the flow and status of nutrients and water, and influence microbial communities in soil and rhizosphere	Gelsomino et al. (1999); Sessitsch et al. (2001); Girvan et al. (2003); Singh et al. (2007); Bach et al. (2010); Chau et al. (2011); Schreiter et al. (2014)
	Soil salinity can affect soil and plant-associated microbial communities	Lozupone and Knight (2007); Yaish et al. (2016); Thiem et al. (2018); Berens et al. (2019)
	Drought can affect soil and plant-associated microbial communities	Bachar et al. (2010); Hueso et al. (2012); Alster et al. (2013); Bogino et al. (2013); Naylor and Coleman-Derr (2018); Xu et al. (2018)
Soil and biotic factors	Distinct microbial communities are correlated with the presence or occurrence of plant pathogens or diseases; suppressive soils could contain more microbes with antagonistic activity; initial differences in the soil microbiome composition can affect plant health	Sanguin et al. (2009); Mendes et al. (2011); Meng et al. (2012); Rosenzweig et al. (2012); Siegel-Hertz et al. (2018); Wei et al. (2019); Zhou et al. (2019)
	Agricultural management practices and land use can cause changes in microbial community composition and diversity	Steenwerth et al. (2002); Garbeva et al. (2006); Lauber et al. (2008); Postma et al. (2008); Reeve et al. (2010); Carbonetto et al. (2014); Peralta et al. (2018); Sun et al. (2018); Le Guillou et al. (2019)

### Soil pH

Soil pH has a strong influence on the solubility and availability of nutrients (Cerozi and Fitzsimmons, 2016), such as carbon (C), nitrogen (N), phosphorus (P), potassium (K), iron (Fe), and zinc (Zn), which most living organisms need for survival and growth (Lindsay, 1995; Tack et al., 1996; Andersson et al., 2000; Wakelin et al., 2008; Griffiths et al., 2011). Accordingly, of all soil properties, the soil pH seems to be the most important factor in affecting soil bacterial diversity and community composition (Fierer and Jackson, 2006; Lauber et al., 2008). In agricultural soils, the bacterial and fungal community structure and catabolic function are also strongly correlated with soil pH (Lauber et al., 2009). Shen et al. (2013) further demonstrated that the bacterial community composition and diversity were strongly affected by pH, but no similar effects are observed for the fungal community. The authors suggest that the strong influence of pH on bacterial community composition but not on fungi may be due to the narrow range of pH for the optimal growth of bacteria as compared with fungi that tolerate a wider pH range. Similar findings indicated that soil pH was the best predictor for bacterial diversity (e.g. highest diversity at near-neutral pH), richness, and community composition, while the soil nutrient status was a stronger driver for the fungal community (Maestre et al., 2015; Zhang et al., 2017; Shen et al., 2018). However, a continental-scale study on dryland sites revealed that the soil pH does not correlate with bacterial diversity, possibly due to higher overall pH values (Li et al., 2017).

### Fertilization and nutrient availability

Since soil pH controls nutrient availability and accessibility and, consequently, changes in bacterial and fungal community structure, then fertilization practices (e.g. chemical versus organic fertilization) and soil amendments must also play an important role. Francioli et al. (2016) showed that a combination of organic and inorganic fertilization led to increased total N and organic C, causing changes to the bacterial community composition, which correlated with taxa involved in organic matter decomposition and nutrient transformation. Goldfarb et al. (2011) found significant differences in the bacterial and fungal communities between mineral, organic, and mineral–organic combined fertilization. Organic fertilization (manure) increased bacterial diversity, stimulating microbial groups known to thrive in nutrient-rich environments, while soils without manure contained microbial groups adapted to nutrient-limited conditions (Eilers et al., 2010). In addition, fertilization altered the relative abundance of plant-beneficial and plant-pathogenic microbes. Overall, pH and total organic C were identified as the major factors driving the structure and activity of the soil microbial community.

One of the most essential nutrients required for cellular metabolism and growth of bacteria is C. Carbon soil amendments can also affect microbial communities (Maestre et al., 2015). Indeed, low molecular weight C amendments, particularly citric acid, of three soil types resulted in shifts in the bacterial communities (Siciliano et al., 2014). However, the responses in these shifts can vary depending on the soil type. Kuramae et al. (2012) concluded that organic C content had a direct positive effect on the diversity and abundance of bacteria and fungi. Further studies showed that other nutrients such as N and P were the major factors influencing bacterial and fungal community structures in the soil and rhizosphere (Marschner et al., 2004).

### Soil water content and temperature

Seasonal cycles and, more importantly, global warming change the temperature, CO_2_ levels, daylight duration, wind, precipitation (rainfall), and/or humidity (Fig. 1). These changes can alter biological and chemical processes in living organisms, such as photosynthesis in plants, or nutrient recycling in the soil (Schuur and Matson, 2001; Borjigidai et al., 2006; Alvarez-Clare and Mack, 2011). Changes in precipitation, temperature, and vegetation as a result of seasonal changes caused shifts in the microbial community structure and function (Habekost et al., 2008; Bell et al., 2009; Koranda et al., 2013). For example, soil moisture had the highest impact on some microbial parameters (e.g. community structure, substrate activity) at the end of winter and the second highest impact at the end of summer (Bell et al., 2009). In addition, other parameters such as microbial biomass and fungal substrate activity highly correlated with temperature in different seasons (Table 1).

Temperature affects microbial growth and activity, and thus can cause shifts in community composition and function (Pettersson and Bååth, 2003; Bell et al., 2009). In some cases, temperature changes of 3 °C resulted in changes in the abundance of fungal and bacterial communities (Bárcenas-Moreno et al., 2009). In contrast, Castro et al. (2010) revealed undetectable effects on cyanobacterial abundance or bacterial community by a 2–3 °C increase in soil temperature, while altered precipitation had significant effects. Taking into account the different experimental settings, it is thus unclear which of the two—water content or temperature—has a larger effect on microbial communities in the soil.

Among a range of climate change drivers (CO_2_, temperature, and precipitation), precipitation had the largest effect on bacterial and fungal community composition (Johnson et al., 2012). By testing the effects of wetting events, Castro et al. (2010) found that the amount of water added had a much greater impact than the irrigation frequency on shaping the bacterial and fungal community structures. In another study, the abundance and community structure of fungi was unaffected by extreme precipitation events compared with more frequent moderate events, which increased bacterial abundance (Frossard et al., 2015). These effects may be due to changes in soil pH or availability of nutrients upon precipitation. For example, the continental-scale study of Zhang et al. (2019) on dryland sites revealed that aridity indirectly affected soil pH and organic C content, leading to reduced diversity and abundance of soil bacteria and fungi.

### Soil type, texture, and structure

Soil type can be a primary determinant of microbial communities because soils comprise a range of characteristics, such as nutrient and water content, cation exchange capacity, or texture and structure. A variety of studies have shown that the soil type can have a strong influence on the soil microbial communities (Girvan et al., 2003; Maestre et al., 2015), as observed in the rhizosphere of grass (*Lolium perenne*) and lettuce (Gelsomino et al., 1999; Singh et al., 2007; Schreiter et al., 2014). The soil texture/structure can affect the size and distribution of particles and pore spaces (Table 1), influencing the flow of water and nutrients and, consequently, lead to changes in the soil microbial community (Girvan et al., 2003; Lauber et al., 2008). Bach et al. (2010) showed that the microbial community differed between silty clay loam and loamy fine sand soil. Chau et al. (2011) observed that soil texture affected bacterial species richness but not bacterial diversity. The microbial community structure was also significantly affected by particle size, whereby higher microbial diversity was attributed to smaller silt and clay particle size than coarse sand fractions (Sessitsch et al., 2001). Moreover, particle size fraction affected the bacterial community structure more than the type of organic soil amendment (Sessitsch et al., 2001).

### Soil salinity and drought: abiotic stresses affecting microbial communities in plants

Plant microbiome studies showed the complex relationship between environmental factors and bacterial community structures, especially in open field conditions, emphasizing the possible bias in laboratory experiments due to the absence of the variability of environmental changes. Extreme environmental changes or abiotic stresses, especially in light of climate change, can cause changes in microbial communities. The soil microbiome can be affected by abiotic stresses both directly (e.g. survival of drought-, salt- or heat-tolerant taxa) (Martiny et al., 2017; Naylor and Coleman-Derr, 2017) or indirectly (e.g. through altered soil chemistry or diffusion rates) (Schimel et al., 2007; Liptzin et al., 2011). Soil salinity and drought are arguably the biggest threats to global food security, and are clearly important factors affecting the structure and dynamics of soil microbiomes and, in turn, the plant microbiota, especially root endophytes. Recently, Berens et al. (2019) demonstrated that salinity treatment, along with leaf age, were crucial factors in determining the microbial community composition in Arabidopsis leaves. The study also identified a leaf age/developmental stage-dependent response to biotic and abiotic stress.

A meta-analysis of soil microbial communities revealed that the global microbial composition in saline soils is more affected by salinity than by any other abiotic factor (Lozupone and Knight, 2007). A significant difference in the endophytic microbial community composition was observed in black alder (*Alnus glutinosa* Gaertn.) roots grown in saline soil, with a decrease in the bacterial diversity and species richness and evenness (Thiem et al., 2018). In date palm (*Phoenix dactylifera*), the endophytic bacterial community in salinity-treated plants contained a higher number of total operational taxonomic units (OTUs) and higher species evenness and diversity, compared with control plants (Yaish et al., 2016). The root microbiome under drought stress conditions is determined by how the stress shapes both the host plant and the surrounding soils, where the total bacterial biomass is reduced (Hueso et al., 2012; Alster et al., 2013). Bogino et al. (2013) demonstrated that the rhizosphere of alfalfa (*Medicago sativa*) plants exposed to differing water-limiting conditions harbor distinct bacterial communities with different abilities to develop biofilms, and thus to establish themselves in this microenvironment. A recent study on the root microbiome of sorghum (*Sorghum bicolor*) demonstrated that drought causes enrichment of a distinct set of roots microbes. The discovery of this drought-induced enrichment and associated shifts in metabolite exchange between the plant and the microbes revealed a potential blueprint for manipulating plant microbiomes for improved crop fitness (Xu et al., 2018).

### Suppressive soil: effects of soil biotic factors on plant health and microbial communities

Soil microbiome studies increasingly focus on improving soil health, quality, and fertility by promoting growth of beneficial while suppressing pathogenic microbes (Schlatter et al., 2017). This is particularly evident when discussing suppressive soils, which are soils that possess the ability to limit the growth and survival of plant pathogens (Baker and Cook, 1974). Suppressive soils fall into two general categories: general disease suppression is attributed to the soil’s total microbiome antagonistic activity against a broad range of soil-borne pathogens, while specific suppression is attributed to an individual taxon or group of microbes and is transferrable by adding pure cultures or small amounts of suppressive soil to conducive (non-suppressive) soil (Weller et al., 2002; Schlatter et al., 2017). The microbial community composition and diversity in soil is important for pathogen suppression, as demonstrated by the lower suppression rates of sterilized or semi-sterilized soil compared with unsterile soil (Garbeva et al., 2006; Mendes et al., 2011; Meng et al., 2012; Svenningsen et al., 2018). Studies have revealed changes in the rhizosphere microbial community upon pathogen infection and identified key taxa that may be involved in suppression of plant pathogens or diseases (Mendes et al., 2011). A 10 year long wheat (*Triticum aestivum*) field study revealed that the outbreak, decline, and suppression of the take-all disease, caused by the pathogenic fungus *Gaeumannomyces graminis* var. *tritici*, was correlated with changes in the rhizosphere bacterial communities (Sanguin et al., 2009). Distinct microbial communities also exist in potato (*Solanum tuberosum*) when comparing common scab-conducive soil, caused by pathogenic bacteria *Streptomyces scabies*, with suppressive soil (Meng et al., 2012; Rosenzweig et al., 2012).

Meta-barcoding analysis of *Fusarium* wilt-suppressive and conducive soils demonstrated that specific genera of fungi were exclusively present, and some bacterial genera were more abundant in suppressive soils (Siegel-Hertz et al., 2018). Zhou et al. (2019) also observed a higher fungal and bacterial richness and diversity in *Fusarium* wilt-conducive than suppressive soils. Furthermore, the type of soil pathogen and soil properties may also play a role in the suppressive potential. Postma et al. (2008) found that, depending on the pathogen, soil suppression correlated not only with specific antagonistic microbes but also with different soil properties. More importantly, a recent study revealed that small initial differences in the soil microbiome composition can affect plant–pathogen interactions and, therefore, plant health under natural field conditions (Wei et al., 2019).

Additionally, agricultural management practices can affect the suppressive potential of soils, primarily due to changes in the microbial communities. Garbeva et al. (2006) revealed a correlation between agricultural management practices on soil microbial community structure and its effect on soil suppression of the pathogenic fungus *Rhizoctonia solani* AG3. Based on crop rotational diversity practices, Peralta et al. (2018) suggested that microbial community composition might be more crucial than microbial diversity in disease suppression.

## Plant-associated microbial communities

Since plant phenotype and fitness depend on the associated microbiome, plants try to recruit the best microbial community under given conditions (e.g. nutrient availability, pathogenic infection, and abiotic stresses). These factors not only affect the plant microbiota but also shape the soil microbiome and, more specifically, the rhizosphere. Hereafter, we will describe different plant-related factors responsible for defining the selected microbial communities (bacteria and fungi) in different plant organs (e.g. seeds, roots, or shoots) (Fig. 1; Table 2).

**Table 2.** Plant factors that are responsible for shaping microbial communities

Factors	Summary	References
Plant factors	Host genotype dictates the development of plant phenotypes and influences the microbial community composition of the rhizosphere, roots, leaves, and seeds	Adams and Kloepper (2002); Lindow and Brandl (2003); Fenner et al. (2005); Berg and Smalla (2009); Delmotte et al. (2009); Aira et al. (2010); Berendsen et al. (2012); Knief et al. (2012); Bálint et al. (2013); Hyung et al. (2014); Barret et al. (2015); Sapkota et al. (2015); Laforest- Lapointe et al. (2016); Müller et al. (2016); Unterseher et al. (2016); Wagner et al. (2016); Gomes et al. (2018); Zhalnina et al. (2018); Jones et al. (2019); Toju et al. (2019); Wassermann et al. (2019)
	Root exudates shape the root and rhizosphere microbial community	Bressan et al. (2009); Hu et al. (2018); Sasse et al. (2018); Stringlis et al. (2018*b*); Cotton et al. (2019); Huang et al. (2019); Voges et al. (2019)
	Nutrient status of P and Fe can alter microbial communities	Castrillo et al. (2017); Stringlis et al. (2018*b*); Finkel et al. (2019); Voges et al. (2019)
	Phytohormones, such as SA and JA, have a role in plant defense responses and can shape microbial community in a host-dependent manner	Pieterse et al. (2009); Carvalhais et al. (2013); Santhanam et al. (2014); Carvalhais et al. (2015); Lebeis et al. (2015); Schlaeppi and Bulgarelli (2015); Carvalhais et al. (2017); Liu et al. (2017 (2018)

### Plant seeds harbor their own microbial community

Seed coating is an efficient tool to deliver beneficial microbes for agricultural applications (Rocha et al., 2019). Interestingly, the seeds of native plants harbor a more specific microbiota than that reported for crop plants, allowing plant populations to survive, persist, and germinate under harsh natural conditions (Fenner et al., 2005; Wassermann et al., 2019). Different studies have investigated the dynamics of the seed microbiota during germination and emergence. Eight plant genotypes mostly affiliated to Brassicaceae were evaluated at three physiological stages: seed, germinating seed, and seedling states (Barret et al., 2015). Similar to bacterial and fungal taxa associated with the rhizosphere and the phyllosphere of various plant species (Toju et al., 2019), the seed microbiota was shown to be composed of three major bacterial phyla, Actinobacteria, Firmicutes, and Proteobacteria, and two fungal classes, the Dothideomycetes and Tremellomycetes (Barret et al., 2015). This suggests that the seeds might serve as a microbial bank for other plant compartments (van Overbeek et al., 2011) where the plants can select for beneficial microbes, which explains the high content of β-Proteobacteria, γ-Proteobacteria, and Firmicutes to suppress diseases (Berendsen et al., 2012). Indeed, bacterial endophytes of maize and rice (*Oryza sativa*) seeds were also found in the root endosphere and rhizosphere of these plants (Johnston-Monje and Raizada, 2011; Hardoim et al., 2012). Notably, Wassermann et al. (2019) conducted a study on seeds of eight native alpine plant species and highlighted the importance of the plant genotype as the main driver of the seed microbial community composition and diversity.

### Plant genotype determines microbiome composition

In the past 20 years, evidence has accumulated that plant genotypes dictate the development of plant phenotypes and influence the microbial community composition of roots, leaves, and seeds (Table 2) (Adams and Kloepper, 2002; Bálint et al., 2013; Sapkota et al., 2015; Müller et al., 2016; Wagner et al., 2016; Adam et al., 2018). For example, Delmotte et al. (2009) investigated the microbiome of different plants of the Fabaceae and Brassicaceae families (e.g. clover, soybean, and Arabidopsis) and highlighted that, despite the ~130 million years of evolutionary divergence between the families (Hyung et al., 2014; Johnston-Monje et al., 2016), ~70% of the phyllosphere microbiota were conserved. This indicates the presence of a large core microbiome with minor host-specific functions of the microbiota. Balint-Kurti et al. (2010) revealed consistent differences among maize genotypes in the diversity of the epiphytic microbial population and identified UV-B-specific loci that genetically correlated with resistance to fungal pathogen infection. The microbes inhabiting the phyllosphere and rhizosphere are affected by the plant species to different degrees due to different plant phenotypic characteristics (Delmotte et al., 2009; Jones et al., 2019). The phyllosphere microbial community is affected by time (day/night) and exclusively by the plant genotype because the compounds secreted in the leaves are limited (Lindow and Brandl, 2003). Sapkota et al. (2015) observed that plant genotype at the species level of cereals (wheat, barley, oat, rye, and triticale) provides 43% of the variance in the total fungal community. By investigating the phyllosphere of five dominant temperate forest tree species (*Acer saccharum*, *Acer rubrum*, *Betula papyrifera*, *Abies balsamea*, and *Picea glauca*), Laforest-Lapointe et al. (2016) demonstrated that host species features, such as wood density and leaf N content, drive the bacterial community structure. Similar results were found for fungal communities in European beech (*Fagus sylvatica*), which were more impacted by leaf physiological characteristics (Unterseher et al., 2016). Moreover, Li et al. (2018) showed that the leaf and root microbiomes of spruce trees grown in a common garden are affected by host genotype, with differences found between the phyllosphere and soil and between bacteria and fungi. Therefore, phenotypic characteristics of the host plant shape the composition of its associated microbial community (Li et al., 2018; Jones et al., 2019).

### Root exudates and their interactions with root-associated microbes

Interactions between plants and their microbial communities are not unidirectional. The host plant provides novel metabolic capabilities to its microbial associates, leading to the adaptation of niche-specialized inhabitants that can have either a positive (mutualist), neutral (commensal), or deleterious (pathogen) impact on plant fitness (Thrall et al., 2007). The rhizosphere is a complex habitat that is surrounded by a soil matrix where the plant roots constantly produce and secrete a diverse suite of metabolites and compounds called root exudates (Knief et al., 2012; Zhalnina et al., 2018). Root exudates are commonly produced with great variation in the chemical composition which is under genetic control of the host (Inderjit and Weston, 2003; Canarini et al., 2019). Root exudates are mainly comprised of primary metabolites such as sugars, amino acids, and carboxylic acids, as well as a diverse set of secondary metabolites (Cesco et al., 2010; Hu et al., 2018).

Root exudates, which represent up to 20% and 15% of fixed C and N, respectively (Haichar et al., 2016; Venturi and Keel, 2016), enrich the soil and rhizosphere and lead to changes in the microbial communities. The rhizosphere community is influenced by both the soil and plant genotype due to differences in root exudate quality and quantity secreted in the soil (Berg and Smalla, 2009; Aira et al., 2010; Gomes et al., 2018). Typically, the quality and quantity are determined by the size, age, and physiological condition of the plant root system. Abiotic stresses can also affect plant root exudates and the microbial community, as shown for citrus plants under salinity and temperature stress (Vives-Peris et al., 2018). Exudates from Macrophylla salt-stressed plants were able to promote the growth of *Pseudomonas putida* KT2440 and *Novosphingobium* sp. HR1a, whereas exudates from Carrizo salt-stressed plants did not promote bacterial growth. Moreover, in the presence of exudates from Macrophylla salt-stressed plants, growth promotion by *Novosphingobium* sp. HR1a was higher than with *P. putida* KT2440, which could be due to the higher tolerance of this strain to salinity stress (Vives-Peris et al., 2018).

Root exudates can also play a role as signaling molecules, attractants, or stimulants in establishing a symbiotic relationships with different microbes and, additionally, function in defense against pathogens (Perret et al., 2000; Kobayashi et al., 2004; Cesco et al., 2010; Baetz and Martinoia, 2014). The growth of soil microbes is usually C limited and the high amounts of sugars, amino acids, and organic acids that plants deposit into the rhizosphere represent a valuable nutrition source for microbial growth (Bais et al., 2006). However, depositing C will attract both pathogenic and beneficial microbes, suggesting that plants not only evolved recognition mechanisms to discriminate between beneficial and pathogenic microorganisms (Passera et al., 2019), but can also change root exudate composition to serve such selective mechanisms. Clear examples are the secretion of communication molecules/attractants such as flavonoids, strigolactones (SLs), or terpenoids (Bais et al., 2006; Venturi and Fuqua, 2013; Massalha et al., 2017). Flavonoids (2-phenyl-1,4-benzopyrone derivatives) are the most important molecules from the symbiotic perspective. Although found throughout the plant kingdom, flavonoids specifically trigger the expression of the rhizobial genes (*nod*, *nol*, and *noe*) required for nodulation and efficient N_2_ fixation of different legume members (Kobayashi et al., 2004; Zgadzaj et al., 2016; Saad et al., 2019). The nodulation capacity varies with flavonoids and rhizobia; and, in some cases, flavonoids may inhibit nodulation (Cooper, 2007; Hassan and Mathesius, 2012). Interestingly, plant fitness determines exudate quality, as seen in non-infected healthy Arabidopsis and rice plants that constitutively produce and release metabolites such as antimicrobial diterpene rhizathalene A or momilactone A to protect plants against infection (Vaughan et al., 2013).

#### The role of root exudates in defense responses

Upon pathogen infection, plants produce low molecular weight antimicrobial compounds, called phytoalexins, that are not detectable in healthy plants (VanEtten et al., 1994). Clear evidence for this comes from *Fusarium graminearum*-infected barley roots, where the infected plant induced the production of antifungal compounds (Lanoue et al., 2010). Glucosinolates are another group of plant metabolites with antimicrobial activities that are specifically produced by Brassicaceae. An Arabidopsis CYP79A1 transgenic line, which produces exogenous glucosinolates, altered the bacterial and fungal communities in the rhizosphere and root tissues (Bressan et al., 2009). The synthetic SL analog GR24 inhibits the growth of an array of phytopathogenic fungi when present in the growth medium (Dor et al., 2011), indicating that secreted SLs can affect natural enemies directly or indirectly by modulating hormonal defense pathways and contribute to below-ground plant biotic stress responses (Torres-Vera et al., 2014). Triterpenes are another group of plant metabolites that possess antifungal and antibacterial activities, suggesting potential roles in shaping the plant microbes (Brown et al., 1963; Papadopoulou et al., 1999; Augustin et al., 2011). Recently, Huang et al. (2019) demonstrated that Arabidopsis produces a range of specialized triterpenes that direct the assembly and maintenance of an Arabidopsis-specific microbiota, enabling it to shape and tailor the microbial community within and around its roots for its own purposes.

#### Root exudates shape the rhizosphere microbiota

Plants also use root exudates to alter the root microbial (bacterial and fungal) communities and exploit them for their own benefits. Maize plants were found to produce and release a mixture of metabolites from the roots, including benzoxazinoids (BXs) such as DIMBOA, which influence the composition of the root-associated microbiota (Hu et al., 2018; Cotton et al., 2019). DIMBOA is relatively short lived and is rapidly converted to the more stable MBOA that accumulates in the soil. As a result, MBOA triggers changes in the structure of the root-associated microbiota in the next plant generation. The microbiota-mediated BX-dependent effects on plant growth and defense were strongly associated with changes in the bacterial, rather than the fungal, rhizosphere community. These changes resulted in increased leaf defense, suppression of herbivore growth, and decreased plant growth, and the latter depended on the plant genetic background (Hu et al., 2018).

Stringlis et al. (2018*b*) provided direct evidence of how a specialized root exudate, the antimicrobial coumarin scopoletin, can cause changes in the microbial community structure and diversity in the rhizosphere. Scopoletin inhibits the fungal pathogens *Fusarium oxysporum* and *Verticillium dahliae* but not the growth-promoting rhizobacteria *Pseudomonas simiae* WCS417 and *P. capeferrum* WCS358. Voges et al. (2019) showed that the lack of coumarin biosynthesis in ‘f6′h1’ mutant lines caused a shift in the root microbial community specifically under Fe deficiency, demonstrating a potential role for Fe-mobilizing coumarins in shaping the Arabidopsis root bacterial community by inhibiting the proliferation of a relatively abundant *Pseudomonas* species via a redox-mediated mechanism.

Overall, the secretion of the root exudates (genotype) leads to chemical changes in the soil composition, soil properties, available nutrients (see below), and toxic elements in the rhizosphere (Neumann and Römheld, 2000; Marschner et al., 2004). All the above studies suggest that molecules derived from these specialized metabolites may play a role in the local adaptation of the plant to the soil environment and microbial ecology. Therefore, the exudation of bioactive compounds in root exudates probably defines the assembly of the plant-specific root and rhizosphere microbial communities for the benefit of the plant.

### Cycling of nutrients between the soil, plant, and associated microbes

Plants are dependent on the growth of soil microbes which possess the metabolic machinery to depolymerize and mineralize organic forms of N, P, K, S, and Fe. In soil, most compounds are bound to organic molecules and are, therefore, minimally bioavailable for plants. To access these nutrients, plants adopt different strategies to interact with their environment for the solubilization and acquisition of nutrients (Lambers et al., 2008; Orwin et al., 2010; Grigulis et al., 2013). These strategies strongly influence plant–microbiota interactions due to the competition between plants and microorganisms for soil nutrients. The impact of plant nutrient resource strategies, plant functional traits, and the diversity of active microbiota through root exudation was studied extensively in the last decade (Guyonnet et al., 2018).

#### Nitrogen, phosphorus, and potassium (NPK)

The relationships between the plant and soil microbiome are governed by the trade-off theory where the plant provides C and, in return, can benefit from essential nutrients provided or facilitated by microbes, such as N, P, and K. For example, different studies highlighted the involvement of N_2_-fixing microbes (free-living ‘non-symbiotic’ or mutualistic ‘symbiotic’) in promoting plant growth (Vitousek et al., 2002; Graham and Vance, 2003; Bahulikar et al., 2014; Gaby and Buckley, 2015). Some bacteria and fungi can solubilize inorganic P or mineralize organic P (Eida et al., 2017; Nehls and Plassard, 2018). Many of these P-mobilizing strains are growth-promoting microbes which can promote plant growth via a wide variety of mechanisms. Thus, it is difficult to correlate P-mobilization mechanisms to the observed growth promotion elicited by these strains (Richardson and Simpson, 2011). However, under P-deficient conditions, plants respond by shaping the root microbial community (Castrillo et al., 2017; Finkel et al., 2019), which could enrich P-solubilizing/mobilizing microbes. Another vital nutrient considered as a key parameter of soil fertility and plant growth is K. As described by Sheng and He (2006), the inoculation of plants by *Bacillus edaphicus* NBT strains increased the production of citric, oxalic, tartaric, succinic, and α-ketogluconic acids, leading to K mobilization from K-containing minerals (e.g. mica and biotite) and chelation of silicon.

#### Iron

Fe is another essential element needed by all living organisms and is considered as a key micronutrient for soil fertility. The combination of the low concentration of Fe^3+^ together with high demand from both plants and microbes leads to a competition for Fe^3+^ in the rhizosphere (Guerinot and Yi, 1994). Bacteria and plants employ different strategies to overcome Fe limitations. For example, different groups of bacteria (e.g. *Pseudomonas*, *Azotobacter*, *Bacillus*, *Enterobacter*, *Serratia*, *Azospirillum*, and *Rhizobium*) produce low molecular weight proteins called siderophores with high affinity to chelate Fe from the soil (Loper and Buyer, 1991; Glick, 2014). Depending on the genotype, plants have adapted different strategies for Fe acquisition, such as the secretion of protons (Guerinot and Yi, 1994), the plasmalemma transport of Fe^2+^ by transporters (Eide et al., 1996; Vert et al., 2002), and/or the reduction of Fe^3+^ to the more stable Fe^2+^ by an NADPH-ferric chelate reductase (Yi and Guerinot, 1996). On the other hand, grasses can synthesize phytosiderophores to form complexes with Fe^3+^ complexes for uptake by specific transporters (von Wirén et al., 2000).

As a part of the adaptive responses to Fe deficiency, plants such as Arabidopsis can produce coumarins: active metabolites that change microbial dynamics by limiting the growth of a plant pathogenic *Pseudomonas* strain (Voges et al., 2019). Moreover, plant iron homeostasis is not only affected upon pathogen infection (Aznar et al., 2015), but also upon root colonization by plant growth-promoting rhizobacteria (PGPRs) (Zamioudis et al., 2015; Verbon et al., 2017). PGPRs are known to trigger induced systemic resistance (ISR) that primes plant tissues for enhanced defense against a broad spectrum of pathogens (Lugtenberg and Kamilova, 2009). A clear connection between ISR and iron homeostasis was demonstrated by Leeman et al. (1996), where the elicitation of ISR against *Fusarium* wilt in radish (*Raphanus sativus*) by beneficial *Pseudomonas* spp. was shown to be more effective under low-iron conditions. Siderophores secreted by *Pseudomonas* spp. were subsequently shown to act as elicitors of ISR in tomato (Meziane et al., 2005) and rice (De Vleesschauwer et al., 2008).

### Phytohormones and their roles in shaping plant microbiota

Plant hormones (phytohormones) play diverse roles in plant physiological processes including mutualistic interactions with soil microbiota (Shigenaga and Argueso, 2016). The well-studied phytohormones are jasmonic acid (JA), salicylic acid (SA) (Boatwright and Pajerowska-Mukhtar, 2013), ethylene (ET) (Ju et al., 2015), abscisic acid (ABA) (Finkelstein, 2013), auxin (Austin et al., 2002), gibberellins (GAs) (Binenbaum et al., 2018), cytokinins (CKs) (Jiang et al., 2013), brassinosteroids (BRs) (Nolan et al., 2017), and SLs (Zwanenburg et al., 2016).

#### Auxin

Indole acetic acid (IAA) plays a role in shaping the microbiome because it regulates the development of lateral and secondary roots, which represent the preferential sites for microbial colonization (Kaldorf and Ludwig-Müller, 2000; Contreras-Cornejo et al., 2009; Zamioudis et al., 2013; Stringlis et al., 2018*a*). Applications of various forms of auxins (IAA, indole-3-butyric acid, 2,4-dichlorophenoxyacetic acid, and 1-naphthaleneacetic acid) promoted the spread of arbuscular mycorrhizal (AM) fungi and arbuscular abundance (J. Liu et al., 2016). The auxin (IAA)-deficient bushy mutant (Symons et al., 1999) showed reduced AM colonization but did not alter AM fungal structures inside the roots (Foo, 2013). Moreover, the tomato auxin-resistant diageotropica (*dgt*) mutant showed lower AM fungal development in both monoxenic and *ex vitro* conditions (Hanlon and Coenen, 2011). On the other hand, different soil microbes, either free-living or plant-associated, produce IAA themselves. Interestingly, 60% of phyllosphere bacteria and 80% of epiphytic bacteria can produce IAA (Spaepen et al., 2007; Kim et al., 2011; Spaepen and Vanderleyden, 2011). The synthesis of IAA and its derivatives was reported for *Acidovorax*, *Agrobacterium*, *Arthrobacter*, *Bacillus*, *Chryseobacterium*, *Enterobacter*, *Pseudomonas*, *Ochrobactrum*, *Mycobacterium*, *Methylobacterium*, and *Stenotrophomonas* species (Omer et al., 2004; Egamberdieva, 2009; Egamberdieva et al., 2015; Eida et al., 2018; Tsolakidou et al., 2019). This large number of bacterial IAA producers suggests that IAA synthesis might be a trait that contributes to survival in the plant environment (Kim et al., 2011). This idea is supported by several reports of different bacteria: IAA mutants of *Erwinia herbicola* (Brandl and Lindow, 1998; Manulis et al., 1998) and *Pseudomonas savastanoi* (Spaepen et al., 2007) showed reduced bacterial proliferation on leaves. Together with the plant endogenous IAA pool, bacterial auxin stimulates plant cell growth and proliferation, as well as plant tolerance to abiotic stresses (Panwar et al., 2016; Sorty et al., 2016; Barnawal et al., 2017).

#### Abscisic acid

Among other functions, ABA is a key regulator of abiotic stress responses. Therefore, ABA-producing bacteria could be selected by plants to promote abiotic stress tolerance. Different soil microorganisms can produce ABA, including several phytopathogenic fungi, such as *Cercospora rosicola*, *C. cruenta* and *Botrytis cinerea* (Zeevaart and Creelman, 1988; Sharon et al., 2007), or bacteria, such as *Azospirillum* (Forchetti et al., 2007; Cohen et al., 2008). Interestingly, bacteria commonly found in the human body, which can live in soil and in water (*Proteus mirabilis*, *P. vulgaris*, *Bacillus megaterium*, *B. cereus*, *Klebsiella pneumoniae*, and *Escherichia coli*), are also capable of producing ABA (Karadeniz et al., 2006).

#### Cytokinin

A number of bacteria produce CKs, such as *Arthrobacter*, *Bacillus*, *Azospirillum*, *Pseudomonas*, and *Methylobacterium* (Naz et al., 2009; Jorge et al., 2019). The CK-producing *B. subtilis* strain IB-22 enhances growth of lettuce and wheat, with high colonization rates throughout the vegetative period and increased wheat productivity (Arkhipova et al., 2006, 2007). Other *B. subtilis* isolates stimulated root biomass of *Platycladus orientalis* by 14% and increased CK levels in leaves by 47% under water stress conditions (Liu et al., 2013). Similar increases of shoot and root biomass were observed in soybean (*Glycine max*) inoculated with *Pseudomonas* and *Arthrobacter* spp. under salinity stress (Naz et al., 2009). *Pseudomonas aurantiaca* TSAU22 and *P. extremorientalis* TSAU6 and TSAU20 enhanced the growth of wheat under salinity stress (Egamberdieva, 2009). Moreover, different members of the *Methylobacterium* genus produce high levels of CKs and increase the tolerance of plants to abiotic stresses (e.g. salt and drought stress) (Knief et al., 2012; Lee et al., 2015; Chanratana et al., 2017; Jorge et al., 2019).

#### Ethylene

A variety of plant processes involve the olefin hydrocarbon ET, including nodulation of legumes by rhizobia (Tamimi and Timko, 2003) and mycorrhizal root interaction (Gamalero et al., 2008). Plants use ET as a regulator of stress responses, such as extreme temperatures, water, UV light, and insect and nematode damage and wounding, as well as in interactions with fungi and bacteria (Abeles et al., 1992). Plant genotype, organ, developmental stage, and the associated microbiota are major determinants of ET signaling and responses (Pierik et al., 2007; Dugardeyn and Van Der Straeten, 2008). Interestingly, more than one-third of all cultivable soil bacteria can produce ET via different pathways (Nagahama et al., 1992). Several plant-associated microbes can increase plant ET levels by 1-aminocyclopropane-1-carboxylate (ACC) synthase (ACS) activity (Suganuma et al., 1995) or produce intermediates, such as KMBA, that cav n be converted to ET *in planta* (de Zélicourt et al., 2018). Plant-associated microbes can also decrease ET levels by producing ACC deaminase, an enzyme responsible for the cleavage of the plant ET precursor ACC into ammonia and α-ketobutyrate. Engineering bacteria with ACC deaminase activity promoted resistance of banana (*Musa* spp.) to *Fusarium* (Liu et al., 2019). ACC deaminase-containing bacteria are relatively common in soil, possibly providing these bacteria with a competitive advantage over other rhizosphere microorganisms by using ACC as an N source (Glick, 2014).

#### Jasmonic acid

JA and its volatile methyl ester, MeJA, play crucial roles in plant defense responses against insects and microbial pathogens (Bari and Jones, 2009). Interestingly, JAs also act as signaling molecules that facilitate interactions between plants and root-associated microorganisms (Pieterse et al., 2009). Current evidence indicates that JA influences the composition of the Arabidopsis root-associated microbiome (Carvalhais et al., 2017). Induction of JA signaling increased the relative abundance of bacterial populations closely related to taxa that are reported to suppress phytopathogens and insects (Schlaeppi and Bulgarelli, 2015). Interestingly, the host genotype determines the effect of JA signaling. For example, JA signaling in rice restricts endophytic colonization by certain N_2_-fixing *Azoarcus* bacterial strains when the host–bacterium interaction is less compatible (Miché et al., 2006) and suppresses nodule formation in the legume *Lotus japonicus* (Nakagawa and Kawaguchi, 2006). On the other hand, JA signaling does not impact the structure of the phyllosphere and root microbiomes of wild tobacco (*Nicotiana attenuate*) (Santhanam et al., 2014). Carvalhais et al. (2013) reported that JA signaling pathways affected the composition of root exudates and rhizosphere bacterial and archaeal communities, and these changes significantly correlated with each other. D cFurthermore, a correlation between root exudate content and the abundance of the bacterial communities was reported by Liu et al. (2017). The authors demonstrated that activation of JA signaling in wheat reduced the diversity and changed the composition of bacterial communities in the root endosphere but not in the shoots or rhizosphere. All this evidence suggests that the changes in root endophyte communities in response to JA signaling may reflect a co-evolved mechanism by which plants recruit microbial symbionts that enhance host biotic stress tolerance (Carvalhais et al., 2015, 2017).

#### Salicylic acid

SA mediates plant defense responses against pathogens (Loake and Grant, 2007; An and Mou, 2014) and establishes beneficial symbioses in legume–rhizobia interactions (Martinez-Abarca et al., 1998). SA has also been shown to modulate the composition of the root microbiota at the family level in Arabidopsis (Lebeis et al., 2015). Depending on the host plant species, different responses of the microbial community were reported for SA. For example, activation of the SA signaling pathway in wheat had no significant impact on the diversity of root-associated microbiomes (Liu et al., 2018). A comparison of the bacterial root microbiome of wild-type Arabidopsis with a set of mutants lacking biosynthesis and/or signaling of SA, JA, and ET (Lebeis et al., 2015) demonstrated clear microbial compositional changes of the root microbiome. Moreover, it was shown that certain bacterial endophytic families may require SA-related processes to colonize the root system. Exogenous application of SA altered the microbial community profile composition in both bulk soil and endophytic compartment samples, indicating SA-mediated selection for microbial families. Moreover, different bacterial strains can use SA in different ways, whether as a growth signal or as a C source. Thus, SA may influence the microbial community structure of the root by ‘gating’ bacterial taxa via a homeostatic control of immune system outputs (Lebeis et al., 2015).

### The plant immune system during beneficial microbe interactions

The plant immune system is a prime microbial target to establish beneficial or pathogenic interactions. Plants can detect both beneficial and pathogenic microbes via pattern recognition receptors that bind microbe-associated molecular patterns (MAMPs), such as chitin for fungi or flagellin for bacteria, triggering a basal defense system to halt the growth of most microbes. This defense mechanism is known as MAMP-triggered immunity (MTI) (Boller and Felix, 2009). Some microbes secrete effector proteins to suppress MTI, allowing successful plant infection via effector-triggered susceptibility (ETS). Another plant defense system relies on plant resistance proteins that can recognize microbial effector proteins to activate effector-triggered immunity (ETI). ETI activates local and systemic responses, such as the SA signaling and expression of pathogen-related (PR) proteins. Activation of systemic acquired resistance (SAR) confers a l ong-lasting protection against a wide variety of pathogens (Glazebrook, 2005).

#### Rhizobial PGPRs: manipulation of the host immune system

Beneficial microbes evolved different strategies to modulate the plant immune system for beneficial association/symbiosis. In legumes, rhizobia evolved different mechanisms to avoid pathogen recognition (Cao et al., 2017). For example, rhizobial flagellin appears to lack the flg22 epitope required for flagellin sensing-2 (FLS2)-mediated MAMP activity (Lopez-Gomez et al., 2012). In contrast to pathogens, the identified rhizobial MAMPs, including flagellin, lipopolysaccharides, peptidoglycans, and K-antigen-type polysaccharides, appear to lack MAMP activity and do not trigger MTI in their hosts (Lopez-Gomez et al., 2012). However, rhizobia induce MTI in the early stages of the infection process in legume roots (Libault et al., 2010). Similar changes in defense-related gene expression patterns were also reported in two other legumes (i.e. *L. japonicus* and *Medicago truncatula*) when inoculated with *Rhizobia* species (Stacey et al., 2006; Jones et al., 2008). For successful invasion and nodule formation, rhizobia can also modulate plant SA levels. Interestingly, when alfalfa plants were inoculated with a non-compatible rhizobium strain, the plants showed increased levels of endogenous SA. However, no changes of SA levels were detected upon inoculation with a compatible rhizobium strain (Martinez-Abarca et al., 1998). Similar results were obtained using a Nod factor synthesis-impaired mutant, indicating the role of Nod factors (host-determined compatibility) in suppressing MTI and SA-triggered responses (Liang et al., 2013).

Similar to pathogens, rhizobium strains manipulate the plant immune system by using effector proteins secreted via type 3 secretion systems (T3SSs), collectively named Nodulation outer protein ‘Nops’ (Marie et al., 2003; Saad et al., 2005; Songwattana et al., 2017). In *Sinorhizobium fredii* strain NGR234, NopL acts as a virulence factor when ectopically expressed in tobacco plants, down-regulating virus-induced PR protein accumulation (Bartsev et al., 2004). Similar to NopL, NopM is involved in the inhibition of plant immunity through misregulation of host mitogen-activated protein kinase (MAPK) activation and by inhibiting reactive oxygen species (ROS) production (Bartsev et al., 2004; Xin et al., 2012). Another effector protein, NopT, induces immune responses and cell death, suggesting the presence of a cognate resistance protein (Dai et al., 2008). The same is likely to be true for the rhizobial effector NopP, as *nop*P mutants in NGR234 showed enhanced nodule formation and lower Pathogenesis-Related 1 (*PR1*) gene expression when inoculated in soybean (Skorpil et al., 2005; López-Baena et al., 2009). T3SS effector suppression of MTI responses is most probably superimposed on the dominant suppressive functions of exopolysaccharides (EPSs) and Nod factors (Zamioudis and Pieterse, 2012).

#### Strategies employed by non-rhizobia PGPRs

Non-rhizobial PGPRs also evolved different strategies to overcome the plant immune system. The presence of T3SSs was also reported in a number of plant-associated PGPR (non-rhizobia) strains including different species of *Pseudomonas* with a potential to synthesize and deliver effector proteins (Loper et al., 2012). *Pseudomonas fluorescens* strains SBW25 and Q8r1-96 have a complete T3SS machinery, and SBW25 secretes multiple effectors including members of the AvrE family (e.g. RopE) (Preston et al., 2001), while Q8r1-96 secretes RopAA of the HopAA1-1 effector family. All the T3SS effectors can suppress typical innate immune responses when ectopically expressed in tobacco (*Nicotiana benthamiana*) (Mavrodi et al., 2011). The supramolecular structure of the T3SS in *P. fluorescens* strain 2P24 was resolved and shown to have retained the ability to secrete effector proteins (P. Liu et al., 2016). The presence of T3SS and potential effector proteins of other beneficial *Pseudomonas* spp. (e.g. *P. simiae* WCS417 and *P. defensor* WCS374) was reported by Stringlis et al. (2019). Effector delivery via T3SS may be one mechanism by which PGPRs can either assist in the suppression of MTI responses or manipulate certain host metabolic processes (Zamioudis and Pieterse, 2012).

PGPRs can activate ISR, which involves both JA and ET signaling pathways, leading to the expression of defense-related genes. Both SAR and ISR are activated for different responses, and, although ISR-mediated protection is less effective, SAR and ISR can also work together to provide the best protection and resistance against pathogens (van Wees et al., 2000). Induction of ISR against a broad range of pathogens through activation of SA-, JA-, or ET-responsive defense-related genes in plants w shown for *Bacillus* spp., *Serratia liquefaciens*, *Penicillium* spp., and *Trichoderma* spp. (Djonović et al., 2006; Hossain et al., 2007; Ongena et al., 2007). In Arabidopsis, the activation of ISR in roots by *Pseudomonas simiae* WCS417r (PGPR) is not accompanied by SA-responsive PR protein gene expression, indicating that WCS417r-mediated ISR functions independently of SA (Pieterse et al., 1996). WCS417 is able to suppress flagellin-triggered MTI responses in Arabidopsis roots via apoplastic secretion of low molecular weight molecules (Millet et al., 2010; Stringlis et al., 2018*a*). By using large-scale transcriptomic analysis and reverse genetics approaches, several components, such as MYB72, β-glucosidase U42 (BGLU42), and MYC2, were shown to be involved in rhizobacteria-mediated ISR (Van der Ent et al., 2009; Zamioudis et al., 2014). Recently, it was shown that the root-specific transcription factor MYB72 plays an important role in rhizobacteria-induced secretion of coumarins that shape the assembly of the microbiome in the rhizosphere, potentially optimizing the association with ISR-inducing rhizobacteria (Stringlis et al., 2018*b*). Other strategies employed by different PGPRs to suppress the root immune system were reported by Yu et al. (2019). For example, *P. capeferrum* WCS358 and *P. simiae* WCS417 quench local Arabidopsis root immune responses by lowering the environmental pH via bacterial gluconic acid (Yu et al., 2019).

Another strategy to modulate the plant immune system by PGPRs is phenotypic variation, where bacteria switch between different morphologies (flagella, lipopolysaccharides, pigmentation, etc.) or change their genetic make-up (Davidson and Surette, 2008; Wisniewski-Dyé and Vial, 2008). A clear example of such a process was observed with *P. brassicacearum* NFM421 (Achouak et al., 2004), which was isolated as a major root-colonizing population from Arabidopsis. NFM421 showed morphological phase variation during root colonization of Arabidopsis, resulting in different colony appearances on agar surfaces. Phase II cells localized to the surface of young roots and root tips, whereas phase I cells localized to root basal parts. The ability of phase II cells to spread and colonize new sites on the root surface correlated with the overproduction of flagellin. Phenotypic variation on plant roots is likely to be a colonization strategy that may explain the high colonization power of *P. brassicacearum* (Achouak et al., 2004) which, similarly to animal pathogens, employs phase variation to avoid detection by the immune system (Kingsley and Bäumler, 2000). Phase variation could also be a strategy of PGPRs to prime the plant immune system. The idea of priming could be explained by the plant–microbiota interaction, where plants discriminate friend from foe and respond by either ignoring, supporting, or eliminating microbes. Furthermore, the response pattern to non-pathogenic bacteria can be determined by the plant genotype (Ofek-Lalzar et al., 2014) and can differ across accessions in their recruitment of *P. fluorescens* (Haney et al., 2015). All these factors must be considered when studying plant-associated microbial communities and selecting individuals for inoculants.

## Synthetic holobiont communities

Recent culture-independent analyses and culture collections have paved the way for developing artificially constructed communities, called synthetic communities (SynComs), for studying plant–microbe interactions and promoting plant growth and health (Lundberg et al., 2012; Bulgarelli et al., 2013; Bodenhausen et al., 2014; Bai et al., 2015; Helfrich et al., 2018; Carlström et al., 2019). SynComs can be assembled by rational bottom-up principles by co-culturing several individual microbes. For example, using a culture-dependent collection from sugarcane (*Saccharum* sp.), Armanhi et al. (2018) designed a SynCom comprised of highly abundant bacterial groups and successfully exploited the SynCom for promoting plant growth (increased biomass) in maize. By genome sequencing and comparative genomic analysis of this SynCom, coupled with colonization experiments, de Souza et al. (2019) found that functions related to nutrient acquisition were enriched in robust colonizers. Lebeis et al. (2015) revealed the importance of SA in shaping the root microbiota. Durán et al. (2018) demonstrated the importance of bacterial root commensals for Arabidopsis survival and biocontrol against filamentous eukaryotes and the importance of bacteria–fungi–oomycete consortia for plant growth promotion. Tsolakidou et al. (2019) used tomato rhizosphere bacteria for designing SynComs that were able to promote tomato growth and suppress *Fusarium* wilt symptoms. Using a bacterial SynCom composed of 185 members, Finkel et al. (2019) showed that excluding *Bulkholderia* isolates from the SynCom resulted in the accumulation of higher phosphate shoot levels in plants under P starvation compared with the full SynCom. Finally, drop-out and late introduction experiments using a SynCom made up of 62 leaf bacterial strains by Carlström et al. (2019) revealed that established microbiota are subject to change by late colonizers. The authors also showed that keystone taxa could play important roles in shaping the community structure, especially of strains that are present at very low relative abundance.

It is important to note that in previously mentioned experiments, the assembly of SynComs was performed by choosing either the most abundant taxa or whole collections based on what was cultured. However, we suggest that the selection should be based on the functional traits and abilities of each SynCom member (e.g. hormone production/modulation, nutrient solubilization, volatile production, colonization abilities, and production of antimicrobial compounds) (Fig. 1). In this way, unique traits of each member can complement each other. Furthermore, functional redundancy of SynCom members can increase the resilience of the inoculants, especially in a complex field system. It is also crucial to determine if the SynCom members are compatible with each other or with the plant and environment.

### Desert plants and endophytic bacteria: a model approach for application of microbial inoculants

Hyper-arid deserts and semi-arid grasslands represent two of the harshest terrestrial environments and occupy >20% of the land surface of Earth. Agriculture in these areas faces many challenges, especially considering climate change-driven increases in temperature and aridity and the detrimental effects of abiotic stresses on crop productivity (Boyer, 1982; Bray et al., 2000). Here, we propose that microbial stimulants, whether single isolates or SynComs, should be selected on the basis of their target environment (e.g. bacteria isolated from salinity-stressed environments to promote salinity stress tolerance in plants). For example, pioneer desert plants or crops grown in semi-arid conditions could serve as a target source for isolating bacterial inoculants or SynComs, which can be exploited for semi-arid agriculture to increase the yield of cash crops (Marasco et al., 2012; Eida et al., 2018). The rhizosphere of drought-sensitive pepper (*Capsicum annuum*), cultivated in the North-Western desert region of Egypt, was enriched in PGPRs with growth-promoting abilities on pepper under drought stress (Marasco et al., 2012). Daur et al. (2018) and de Zélicourt et al. (2018) showed that bacterial strains isolated from the rhizosphere and endosphere of desert plants, respectively, in Saudi Arabia were able to boost the yield of alfalfa plants under desert agricultural conditions. The endophytic bacterium *Enterobacter* sp. SA187 from one of these collections survives under abiotic stresses and has PGPR traits (Andrés-Barrao et al., 2017). Application of SA187 was successful in field trials with alfalfa using low and high saline irrigation under desert conditions (de Zélicourt et al., 2018). The success of transferring beneficial microbe-induced abiotic stress tolerance from the lab to the field was probably because the field trials were performed in a similar environment to that from which the bacteria were isolated.

In an effort to achieve sustainable agriculture on semi-arid land, the DARWIN21 project (http://www.darwin21.org/) provides a database of bacterial strains isolated from pioneer desert plants native to the Middle East deserts (e.g. Jordan, Saudi Arabia, and Pakistan). Specific strains showed a great potential for desert agriculture (Bang et al., 2018; de Zélicourt et al., 2018; Bokhari et al., 2019), and draft genome sequences of some of these bacterial isolates have been released (Lafi et al., 2016a, b, c, 2017a, b, c, d), in addition to complete genome sequence analyses (Andrés-Barrao et al., 2017; Eida et al., 2020). We suggest that root-associated microbiota isolated from plants living in extreme conditions, possibly due to evolutionary selection, are ideal for obtaining plant growth-promoting microbes with traits for plant growth and promotion of abiotic or biotic stress tolerance.

## Limitations of microbial community experiments and their applications in agriculture

Plant beneficial microbes become increasingly important for application in agriculture, primarily due to the significant effects of indigenous microbial communities on plant growth and health and the possibility of engineering microbiomes to control plant traits and produce antimicrobial compounds (Mueller and Sachs, 2015; Gopal and Gupta, 2016; Helfrich et al., 2018; Herrera Paredes et al., 2018). However, to understand the molecular and ecological functions of individual members in host-associated microbiomes is a major scientific challenge. This is due to the high complexity and genetic diversity at the species level in microbial communities, including the changing abiotic and biotic factors that dramatically structure microbial communities and the limitations in culturability of many microbes and in nucleic acid-based ‘omic’ approaches (Curtis et al., 2002; Morales and Holben, 2011). Furthermore, the lack of both systematic and comprehensive microbial culture collections for reconstruction experiments and model organisms for understanding plant–microbe interactions limits the progress in this field.

### Culture-dependent community analysis and culture collections

Many studies demonstrate the limitations of culture-dependent community analysis when compared with culture-independent approaches. Two main problems arise when comparing these two methods: culturability and presence of rare taxa. Only a small fraction of the bacterial community can be cultured and those microbes often occur at very low abundance (Sogin et al., 2006; Pereira et al., 2011; Yashiro et al., 2011; Shade et al., 2012; Lee et al., 2016; Eida et al., 2018). The detection of rare species in culture-independent approaches depends on the sequencing technology or, more specifically, the sequencing depth and quality, the amplicon size, and primer pairs (Hiergeist et al., 2015; Beckers et al., 2016). Furthermore, DNA extraction and marker gene sequencing often do not discriminate between intracellular DNA from intact cells and extracellular DNA from lysed or dead cells (Nielsen et al., 2007). Challenges in culturability arise due to several reasons: (i) different species require different growth media and/or fastidious growth conditions; (ii) some microbes are obligate endophytes and need a host to survive; (iii) fast-growing or antagonistic microbes can constrain or inhibit growth of slow-growing strains; and (iv) growth or dominance of some species relies on the presence of others (Vartoukian et al., 2010; Yashiro et al., 2011; Niu et al., 2017; Sarhan et al., 2019).

### Microbial community studies under natural, field, and laboratory settings

There are also other limitations in understanding community changes under natural or field settings. First, natural or field settings contain multiple interdependent factors that cannot be controlled. The soil properties, biological components, and climate all converge, giving rise to a complex environment where a certain microbial community structure is formed and where the root microbiota’s function could be affected (Fig. 1). Any change in one factor could affect all others, leading to false correlations as to which determinant factor caused changes in the community. For example, Bárcenas-Moreno et al. (2009) reasoned that changing soil pH would introduce changes in several other factors, making it difficult to separate pH from the other effects on soil. Often, comparing one factor (e.g. soil pH) from different natural soils can introduce further problems due to the presence of other factors (e.g. soil nutrients) which may play important roles in shaping the microbial community. Experimenting on microbial communities using single factors is only possible under laboratory conditions allowing an understanding of how each component of the soil environment plays a role in changing the microbial communities.

### Technical aspects of community experiments

The sampling method, such as taking soil samples from different depths, can also lead to variable conclusions. For example, the bacterial and fungal communities differ depending on soil depth (topsoil versus subsoil) over long-term fertilization studies (Gu et al., 2017). Many studies have shown that the microbial diversity typically decreases with soil depth, probably owing to the decreased exposure to fertilizers from topsoil to subsoil (Li et al., 2014; Feng et al., 2019). Sampling of bulk soil can introduce high variability and, therefore, it is important to take into consideration sampling strategies to account for this variability (Ogram et al., 2007). Similarly, sampling plant tissue (e.g. leaves) of different age or developmental stage could introduce variability (Fig. 1).

The efficiency of genomic DNA extraction and the number of 16S rRNA copies per cell can vary depending on the bacteria (Frostegård et al., 1999; Klappenbach et al., 2000; Shrestha et al., 2007; Ketchum et al., 2018). Therefore, obtaining accurate abundances of each bacterial strain without knowing the number of 16S rRNA copies within their genomes is an additional limitation. Furthermore, contamination of samples, whether during sampling or during library preparation, can give rise to sequences not representative of the reality (Tanner et al., 1998). Finally, human factors such as agricultural management practices and land use can also affect microbial communities and soil health, and thus should also be considered when performing community experiments (Fig. 1) (Steenwerth et al., 2002; Lauber et al., 2008; Reeve et al., 2010; Carbonetto et al., 2014; Peralta et al., 2018; Sun et al., 2018; Le Guillou et al., 2019).

## A roadmap for successful applications of plant-associated microbial inoculants

**Fig. 2.**
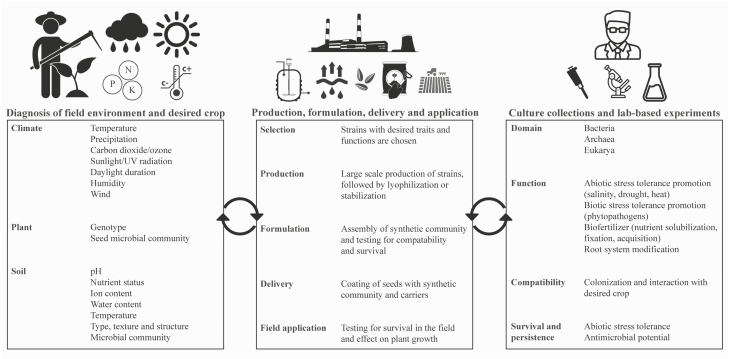
Proposed framework for the successful application of microbial inoculants in agriculture. A framework in which the farmers/farming industry, scientific community, and research and agricultural technology companies collectively contribute to reach the goal of successful microbial inoculant applications. Microbial inoculants must be customized for the target crop, climate, and soil properties (left panel). An increase in scientific research of plant microbiomes, culture collections, and functional characterization of potential microbial inoculants paves the way for meeting farmers’ requirements (right panel). The integration of available microbial inoculants with farmers’ requirements and the large-scale production and formulation (especially for SynComs) is performed by agricultural technology companies (center panel). Collaboration and constant feedback between all three entities is required for the success of field application.

The construction and application of customized inoculants serve an important purpose for enhancing sustainable agriculture by increasing crop health and productivity. Microbiome studies and application of SynComs would greatly advance our knowledge of plant–microbe interactions when complemented with efforts to study and develop model systems from these synthetic communities. As discussed earlier, microbial community structure, function, and composition largely depend on the plant host/genotype, soil properties, the indigenous microbial community, and abiotic factors (Fig. 1; Tables 1, 2). Thus, there are many limitations and challenges of applying microbial inoculants in real, large-scale agricultural field settings. Here, we propose a framework in which the farmers, scientific community, and agricultural technology companies collectively contribute to reach the goal of successful microbial inoculant applications (Fig. 2).

The first aspect in this framework is the thorough analysis of the target field environment and crop of interest (Fig. 2, left panel). Due to the presence of many factors that could affect microbial communities, the inoculants have to be customized to the target crop, field, environmental conditions, and agricultural management practices. Here, a characterization and solid understanding of the climate of the geographical location of the field (e.g. temperature, annual precipitation and humidity levels, and wind speeds) and soil properties (e.g. pH, nutrient status, moisture content, temperature, and microbial community) is performed. Then, the choice of crop plant and its genotype/variety are determined, specifically based on compatibility with climate/soil and economic feasibility. This also requires analysis of the indigenous seed microbial community as it may interfere with the applied inoculants.

The second aspect is a cornerstone in this framework and is pivotal for the success of inoculant application over a wide geographical context. This step requires an increase in scientific research on plant microbiomes and lab-based experiments as well as more culture-dependent isolations. Culturability of environmental microbes can be challenging, therefore its increase will require clever integration of omics (e.g. meta-transcriptomics) and novel culturomics techniques (e.g. plant-based media) (Bomar et al., 2011; Kwak et al., 2018; Sarhan et al., 2019). More importantly, characterization of the single isolates’ functions, survival abilities, plant growth-promoting traits, growth/stress tolerance-promoting mechanisms. and their compatibility with the desired crop is crucial (Fig. 2, right panel). Efforts to achieve this require the increase of whole-genome sequencing, functional characterization of isolates, plant phenotyping, and application of meta-omics (genomics, transcriptomics, proteomics, and metabolomics) approaches, in addition to developing sequencing technologies, bioinformatics models, and tools (Grosskopf and Soyer, 2014; Levy et al., 2018; Sergaki et al., 2018; Marco and Abram, 2019). For example, Rodrigues et al. (2018) recently developed a user-friendly web tool that makes use of the large amounts of microbiome data sets to identify the core microbiome associated with different habitats. Cross et al. (2019) used a reverse genetics approach to isolate and cultivate previously uncultured bacteria. Carper et al. (2019, Preprint) developed advanced computational programs that could overcome the limitations of amplicon sequencing in distinguishing members of a diverse community while maintaining desired member attributes.

The formation of systematic culture collections that cover a broad range of microbial domains (e.g. bacteria, archaea, and eukaryotes) must be considered (Fig. 2, right panel). Although most microbiome studies take into account bacteria and fungi, other microorganisms are also present in soil and could interact, symbiotically or antagonistically, with plants. For example, a recently isolated ammonia-oxidizing archaeon can promote the growth of Arabidopsis and induce systemic resistance against necrotrophic and biotrophic bacteria (Jung et al., 2016; Song et al., 2019). Indeed, archaea are important players in plants (e.g. rice), and their community composition responds to changes (e.g. plant aging and development) or stresses (e.g. drought) (Erkel et al., 2006; Edwards et al., 2018). Interestingly, bacteriophages have been recently shown to control soil-borne pathogens and thus should not be disregarded as a factor in selecting inoculants (Wang et al., 2019). Therefore, future community experiments and culture-dependent isolation should not disregard the presence of other microbial players, which could provide a clearer picture of the complex nature of microbiomes and improve their field application.

The third aspect of our proposed framework is the integration of the field data from farmers and available microbial resources from scientific research by agricultural technology companies in order to customize a suitable inoculant for the prescribed purpose (Fig. 2, center panel). First, the strain(s) are selected from the culture collections based on the desired traits and function, which are determined by field analysis. Large-scale production of the strains is then needed, followed by other processes, such as lyophilization for long-term storage and transport. For formulation of inoculants, the assembly of SynComs and testing the compatibility and survival of each SynCom member with each other is necessary. The single strain or SynComs inoculants can then be delivered for field testing either as lyophilized powder or by coating of seeds of the target crop. Finally, the performance of the inoculant in a field setting similar to the target field/environment is evaluated.

Finally, the framework relies on the constant feedback between all three aspects. Additionally, the increase in soil and plant microbiome data and development of models could assist in predicting how SynComs respond, adapt, and/or survive in the target environment and crop plant. The increase in funding for plant–microbiome research and formation of policies for the use of microbial inoculants in different countries are also needed to be considered for the overall success of achieving global food security in the future.
